# Dopamine-mediated calcium channel regulation in synaptic suppression in L. stagnalis interneurons

**DOI:** 10.1080/19336950.2018.1457897

**Published:** 2018-05-08

**Authors:** Nancy Dong, David W. K. Lee, Hong-Shuo Sun, Zhong-Ping Feng

**Affiliations:** Department of Physiology, Faculty of Medicine, University of Toronto, Toronto, ON, Canada

**Keywords:** dopamine, interneuron, synaptic output, voltage-gated calcium channels

## Abstract

D2 dopamine receptor-mediated suppression of synaptic transmission from interneurons plays a key role in neurobiological functions across species, ranging from respiration to memory formation. In this study, we investigated the mechanisms of D2 receptor-dependent suppression using soma-soma synapse between respiratory interneuron VD4 and LPeD1 in the mollusk *Lymnaea stagnalis (L. stagnalis*). We studied the effects of dopamine on voltage-dependent Ca^2+^ current and synaptic vesicle release from the VD4. We report that dopamine inhibits voltage-dependent Ca^2+^ current in the VD4 by both voltage-dependent and -independent mechanisms. Dopamine also suppresses synaptic vesicle release downstream of activity-dependent Ca^2+^ influx. Our study demonstrated that dopamine acts through D2 receptors to inhibit interneuron synaptic transmission through both voltage-dependent Ca^2+^ channel-dependent and -independent pathways. Taken together, these findings expand our understanding of dopamine function and fundamental mechanisms that shape the dynamics of neural circuit.

## Introduction

Dopamine (DA) regulation of neural circuit function is ubiquitously observed across species. Acting through D1 and D2 classes of G protein-coupled receptors (GPCRs), DA broadly regulates synaptic transmission in the central nervous system (CNS) through modulation of presynaptic neurotransmitter release and postsynaptic response [92]. In particular, DAergic modulation of synaptic output from interneurons, through the activation of D2 receptors, modulates circuit function in a wide range of neurobiological functions, from fundamental homeostatic processes to higher order neural function. For example, in the freshwater pond snail *Lymnaea stagnalis*, rhythmic DAergic inhibition of respiratory interneuron output is key to mediating aerial respiration behaviour [88,75]. In the mammalian brain, DAergic D2 receptor-dependent inhibition of neurotransmitter release from interneurons in the striatum [21,23,53] and amygdala [10,19,72] are critically involved in motor control and associative learning. Therefore, understanding the mechanisms underlying D2 receptor-mediated DAergic inhibition of interneuron synaptic output is of particular physiological significance.

Activation of D2 receptors inhibited voltage-gated Ca^2+^ current (*I*_Ca_) in both *L. stagnalis* [4] and mammalian [99,16,74] interneurons. As activity-induced Ca^2+^ influx is a key determinant of presynaptic vesicle release [104], this likely contributes to DAergic inhibition of interneuron synaptic output. D2 receptor-mediated inhibition of voltage-gated Ca^2+^ channels occurs through both voltage-dependent and -independent mechanisms [15,15,60]. The voltage-dependent mechanism involves direct binding between the G-protein βγ subunit and the α1 subunit of voltage-gated Ca^2+^ channels [20,37,79,101], which results in reduction of the peak current amplitude and slowing of current activation kinetics [71,2,5,31]. The voltage-dependence stems from the ability of a strong depolarization to induce unbinding of the Gβγ subunit and relieve part of the inhibition [5,13,30,42,57]. In contrast, the slower and longer-lasting voltage-independent inhibition can be mediated through a variety of mechanisms, including regulation of voltage-gated Ca^2+^channel surface expression levels through direct interaction with GPCRs [60], involvement of the Gα subunit [51], soluble second messenger cascades [49,38] and kinase activation [80,15,25]. However, whether D2 receptor-mediated inhibition of voltage-gated Ca^2+^ channels leads to synaptic suppression in interneurons remains unclear.

The central pattern generator network that gives rise to the aerial respiration behaviour in the mollusk *Lymnaea stagnalis* provides a well-characterized and highly tractable model for examining DA-mediated regulation of voltage-gated Ca^2+^ channels and synaptic output in interneurons. This circuit consists of the DAergic pacemaker neuron RPeD1 and the follower interneurons VD4 and IP3i [96]. DAergic input from the RPeD1 to the follower cells is necessary for rhythmic network activity of the CPG, as when VD4 and IP3i are cultured together in the absence of the RPeD1, pulsatile application of DA elicited a series of alternating bursts similar to that exhibited by the intact three-interneuron network both *in vivo* and *in vitro* [88]*.* In a well-established *in vitro* soma-soma synapse model [34], VD4 forms an excitatory cholinergic synapse with its postsynaptic target LPeD1 [45,97,98]. The soma-soma synapse between VD4 and LPeD1 allows direct access to both pre- and post-synaptic compartments for electrophysiological, pharmacological, and imaging studies. Furthermore, DA receptors, G protein subunits and Cav2 channels have been well-characterized in the *L. stagnalis* CNS. Both D1- and D2-like receptors have been identified in *L. stagnalis* central neurons [26,48]. The effects of DA on the VD4 and IP3i are shown to be mediated through D2-like receptors, as the effects can be blocked only by the D2 receptor antagonist, sulpiride [68,69].

In this study, we employed the VD4 to LPeD1 synapse model to study the endogenous mechanisms underlying DAergic modulation of voltage-gated Ca^2+^ channels and synaptic output in interneurons. We found that DA not only inhibits *I*_Ca_ in the VD4 through both voltage-dependent and independent pathways, but also suppresses synaptic vesicle release downstream of voltage-gated Ca^2+^ channel-conducted Ca^2+^ influx.

## Materials and methods

### Sequence alignment and phylogenetic analysis

Nucleotide sequences of the *L. stagnalis* CNS transcriptome assembly [82] were retrieved from the European Nucleotide Archive (https://www.ebi.ac.uk/ena) (accession numbers FX180119- FX296473). Open reading frames (>100 amino acids) were identified using TransDecoder v5.0.2 [44] to form the predicted protein-coding sequence set.

Protein sequences of vertebrate orthologs of D2 subfamily receptors, G protein subunits and Cav2 channels were retrieved from UniProt [91] (Table 1). For each protein, sequences of the orthologs were aligned via MUSCLE [29] to create a profile HMM (HMMER v3.1b1) [28] that was then used to query the predicted protein-coding sequences of the *L. stagnalis* CNS transcriptome (cut-off E-value:1e-5). The top hit was searched against the Nr database using BLAST [1] to confirm protein identity. The *L. stagnalis* sequences were aligned with their respective vertebrate orthologs using MUSCLE and analyzed in MEGA7 (Kumar et al., 2016) to find the best-fit model and construct the phylogenetic tree with 500 bootstrap replicates. Functional protein domains were identified according to UniProt annotations.

**Table 1.** Accession numbers of proteins used to construct profile HMM and sequence alignments.

Gene	Human	Non-human primate	Rat	Mouse	Vertebrate fish	*Lymnaea*
Cav2.1	O00555	H2NXR5	P54282	P97445	F8W3Z0	FX182534
Cav2.2	Q00975	H2PU57	Q02294	O55017	A0A146UZM5	
DRD2	P14416	H2NFB8	P61169	P61168	Q8AWE0	
DRD3	P35462	H2P9P0	P19020	P30728	Q6DGJ2	FX186271
DRD4	P21917	H2NCB0	P30729	P51436	Q5DJ15	
GNAO	P09471	H2NQY0	P59215	P18872	F8W442	FX182600
GNAI1	P63096	Q5RAD4	P10824	B2RSH2	Q7T3D3	
GNAI2	P04899	H2QMN4	P04897	P08752	Q6TNT8	FX180365
GNAI3	P08754	H2PZK9	P08753	Q9DC51	A9JTC8	
Gβ_1_	P62873	Q5R5W8	P54311	P62874	Q6PH57	FX180485
Gγ_2_	P59768	Q5R7U4	NP_001244278.1	P63213	Q15KE4	FX191281

### Animals

Freshwater pond snails, *L. stagnalis*, obtained from an inbred culture at the Free University, Amsterdam, were raised and maintained in aquaria at the University of Toronto. All animals used were kept in water at room temperature (20–22°C) on a 12 hr light/dark cycle and fed lettuce twice a week (Monday and Friday) and Purina Trout Chow once (Wednesday). One- to two-month-old snails with shell length of 10–15 mm were used in all experiments.

### Dissection

All animals were dissected under sterile conditions. Snails were anaesthetized for 10 minutes in ∼10% (v/v) Listerine in water. The anaesthetized snails were then de-shelled with fine curved forceps and pinned to a black silicone rubber–based dissection dish containing normal snail saline (NSS) (mM: 51.3 NaCl, 1.7 KCl, 4.1 CaCl2, and 1.5 MgCl2, 5.0 HEPES, pH adjusted to 7.9 with NaOH). A dorsal midline incision was made to expose the CNS, including the central ring and the buccal ganglia from under the buccal mass. The CNS was subsequently removed from the animal using fine dissection scissors and immediately placed in the antibiotic-containing solution (ACS): NSS containing 50 mg/mL gentamycin (Sigma, ON, Canada).

### Cell culture

Conditioning medium (CM) was prepared by incubating 8-12 acutely isolated central ring ganglia in 6 ml defined medium (DM) (mM: serum-free 50% (v/v) Liebowitz L-15 medium (GIBCO, NY, USA) containing 40.0 NaCl, 1.7 KCl, 4.1 CaCl2, 1.5 MgCl2, 5.0 HEPES, 10 glucose, 1.0 L-glutamine, 20-mg/mL gentamycin, pH adjusted to 7.9 with NaOH) in glass Petri dishes pre-coated with Sigmacote (Sigma) for 72 hrs, as described previously [81,34,89]. The resulting 1X CM was frozen in polypropylene tubes at −20°C until required. Subsequently, the brains were then rewashed in ACS and then re-incubated in DM for another 3–5 days to produce 2X CM. A further session of washing and re-incubating for another week produced 3X CM.

For cell isolation, the central ring ganglia were treated with trypsin (Type III, 3 mg/mL, Sigma) dissolved in NSS for 20 min to weaken the outer glial sheath surrounding the central ganglia. The brains were then treated with trypsin inhibitor (3 mg/mL, Sigma) dissolved in NSS for 18 min. Upon completion of digestion, the ganglia were then placed in CM on a black silicone rubber based dissection dish. The connective tissue sheath surrounding the neuronal ganglia was removed using fine forceps. The VD4 and LPeD1 neurons were identified by colour and size in the visceral ganglia and left pedal dorsal ganglia, respectively. Gentle suction via a 2mm fire-polished pipette (World Precision Instruments, FL, USA) that was coated with Sigmacote (Sigma) was used to isolate the identified neurons. The neurons were subsequently plated onto a poly-L-lysine coated glass bottom culture dish filled with pre-prepared CM.

For single cell configuration, acutely isolated VD4 neurons were plated in culture dishes independently from one another for 24 hrs prior to testing. To culture synaptic pairs, acutely isolated VD4 and LPeD1 neurons were plated in culture dishes in close proximity to each other to allow for direct contact between respective neurites. Neurons were kept in culture for 24 hrs to allow for synaptic formation prior to experiments. The formation of the synapse was confirmed by electrical analysis through noting the existence of a postsynaptic response correlated to presynaptic electrical stimulation.

### Electrophysiology

As previously described electrophysiological recordings were performed under current clamp mode [34,33,39], or voltage-clamp mode [33,52,65], using an Axopatch 700A amplifier (Axon Instruments, CA, USA) connected to an analog-to-digital interface Digidata 1322 that was linked to a personal computer running pClamp9 (Axon Instruments) [39,65]. Recording pipettes were pulled using a Flaming-Brown micropipette puller (Model P-87, Sutter Instruments, Novato, CA, USA. The data were filtered at 1 kHz (-3 dB) using a 4- pole Bessel filter and digitized at a sampling frequency of 2 kHz. Data was analyzed using Clampfit version 9.2 (Axon Instruments). All curve fittings were carried out using Origin 7 (OriginLab, MA, USA).

### Whole-cell (ruptured) patch-clamp recordings

Pipettes were filled with whole cell recording pipette solution (mM: 29 KCl, 2.3 CaCl2, 2 MgATP, 0.1 GTP-Tris, 11 EGTA, 10 HEPES, pH adjusted to 7.4 with KOH) (series resistances ∼1 – 2 MΩ). Whole cell recordings were carried out in modified NSS (mM: 10 CaCl2, 45.7 TEA-Cl, 1 MgCl2, 10 HEPES, 2 4-AP, pH adjusted to 7.9 with TEA-OH). Dopamine (DA) (Catalogue #62-31-7, Sigma) was applied via perfusion in the bath solution. Based on previous report that the maximum effective dose of DA on VD4 interneurons is 100 µM [4], we had chosen 10 µM DA for all experiments, unless noted otherwise, to ensure consistency of response.

For voltage step experiments, currents were elicited by stepping from the holding potential to the test potential of +30mV for 50 ms. A prepulse protocol was applied consisting of a holding potential of −70 mV which first stepped to a depolarization of +80 mV for 10 ms, then to test voltage of +30mV for 50 ms separated by an inter-pulse voltage of −70 mV for 5 ms (Figure 7A). To generate the current-voltage (IV) curve, the holding potential was set to −70 mV and 50 ms depolarizing steps from −40 mV to +100mV with a +5mV interval was used. The IV relation was generated from peak inward current using a Boltzmann fit (I=G*(V-Vrev)*(1-(1/(1+exp((V-Vh)/S))))).

To generate the AP waveform (APW), single VD4 neurons were current clamped via a sharp intracellular pipette in NSS bath solution to evoke and record APs endogenous to those neurons. Positive current was injected at levels to induce and maintain an AP train at 2Hz. This frequency was chosen because it represents a sustainable firing rate composed of single APs. The averaged AP profile showed a threshold voltage of −29 mV, an overshoot voltage of +35mV, and half width of 4.5 ms (Figure 6A). Experiments conducted using the APW was controlled using pClamp9. The APW was flanked with the holding potential of −70 mV. Data acquisition was filtered at 1 kHz (-3 dB) using a 4- pole Bessel filter and digitized at a sampling frequency of 2 kHz. Leak currents were subtracted via P/N leak subtraction protocol using 4 positive subsweeps from a holding level of −70 mV. Data was analyzed using Clampfit version 9.2. All curve fittings were carried out using Origin 7.

### Simultaneous pre- and post-synaptic monitoring of VD4-LPeD1 synaptic transmission

To study synaptic transmission between the VD4 and LPeD1 neurons, a dual clamp protocol was used (Figure 4B). The presynaptic cell was current clamped via the intracellular sharp electrode and the postsynaptic cell was simultaneously voltage clamped via the patch electrode. Prior to testing, the presynaptic neuron was kept hyperpolarized to −70 mV by negative current injection to prevent any unwanted firing of APs. During testing, APs were induced by positive current injection to depolarize the neuron just past its firing threshold. A 5s long 1 Hz current pulse train was used to elicit five APs. Postsynaptic responses were detected as excitatory postsynaptic currents (EPSCs). The existence of a functional synapse was confirmed when an EPSC was correlated with a presynaptic AP. All recordings were carried out in NSS bath solution.

### Ratiometric FURA-2 Ca^2+^ imaging

Intracellular Ca^2+^ ([Ca^2+^]_i_) was measured using a FURA-2 ratiometric Ca^2+^ imaging system, as described previously [33]. For intracellular sharp recording experiments, the cell-permeant AM-FURA-2, was used. The cells were incubated with 10 μM AM-FURA-2 (Molecular Probes, OR, USA) for 30 minutes and washed out with recording solution three times prior to imaging. The experiments were carried out in the dark to prevent photobleaching of the dye. The cells were stimulated with simultaneous intracellular recording, and the corresponding FURA-2 fluorescence signal was excited at 340 and 380 nm wavelengths generated by UV light from a 100W Hg/Xe-arc lamp, alternately passed through 340 and 380 nm excitation filters using a high speed random monochromator controlled by Image Pro 5 (Photon Technology International). The fluorescence signal, generated after the excitation light, was reflected via a 430 nm dichroic mirror, passed through a 530 nm long-pass emission filter, and detected and digitized by an intensified charged-coupled device (ICCD) camera (Photon Technology International) in Image Pro 5. Signal acquisition was done in hi-speed acquisition mode using a 10 ms exposure time per excitation wavelength at 8x8 binning. Intensity of fluorescent signals detected at 340 and 380 nm were then processed to floating point images before being used to generate Poenie-Tsien ratio intensity images. These images were used for all FURA-2 analyses.

For voltage clamp experiments, the cell impermeant FURA-2 penta-potassium salt was loaded into neurons through the patch pipette by inclusion in the pipette solution. A final concentration of 100 µM FURA-2 penta-potassium salt was used and allowed to dialyze into the neuron after establishing a seal and rupture.

### FM1-43 imaging of vesicle release

Levels of vesicle release were monitored using the styryl dye N-(3-Triethylammoniumpropyl)-4-(4-(Dibutylamino) Styryl) Pyridinium Dibromide (FM1-43) (Molecular Probes). This water-soluble dye fluoresces when it binds to a lipid-rich membrane and has been commonly used to visualize synaptic vesicles following endocytotic processes. FM1-43 dye (4 µM) was added to culture dishes and allowed to equilibrate for 5 minutes. To load the dye, neurons were induced to fire APs by intracellular sharp electrode current injection. Two 5s current pulses separated by 30s were used, which caused endocytosis of the FM1-43 dye. Any dye that was not endocytosed was subsequently washed away by exchanging NSS bath solution once every 5 minutes for a total of three washes. The experiments were carried out in the dark to prevent photobleaching of the dye. Exocytosis of FM1-43 dye and subsequent destaining of dye was achieved by either electrical stimulation or ionomycin application. Electrical stimulation of vesicle release was performed using intracellular electrodes to inject current pulses into FM1-43 loaded neurons, to cause unloading of dye. The current injection protocol consisted of three current injection pulses of 5s each separated by 30s, which was controlled by pClamp9 software. Ionomycin stimulation of vesicle release was performed by rapid bath exchange of 2 μM ionomycin dissolved in NSS bath solution onto neurons.

Imaging of FM1-43 fluorescence was acquired simultaneously with the above mentioned stimulus protocols. FM1-43 fluorescence signal was excited at 488 nm wavelength generated by UV light from a 100 W Hg/Xe-arc lamp, alternately passed through a 488nm excitation filter, which was controlled by Image Pro 5. Emitted light was passed through a band pass filter 510 nm–560 nm and detected and digitized by an intensified charged-coupled device (ICCD) camera in Image Pro 5. Signals were sampled at 0.5Hz using a 300 ms exposure time and 4×4 binning.

Fluorescent intensities in single neurons were analyzed using the averaged fluorescence from the entire soma. In synaptically paired neurons, fluorescence intensities were taken from three regions: (i) presynaptic contact site (CS), (ii) presynaptic non-contact site, (iii) postsynaptic non-contact site (Figure 4E).

### Data analysis and statistics

Unless otherwise stated, data are presented as the mean ± s.e.m. Statistical analyses were carried out using SigmaStat 3.0 (Jandel Scientific, IL, USA). Differences between mean values from each experimental group were tested using a paired t-test for two groups or one-way analysis of variance (ANOVA, Holm-Sidak or Tukey method) followed by Holm-Sidak post-hoc test for multiple comparisons. Differences were considered significant if *p* < 0.05.

## Results

### Identification and phylogenetic analyses of *L. stagnalis* orthologs of D2-like receptors, G protein subunits and Cav2 channels

**Figure 1.**
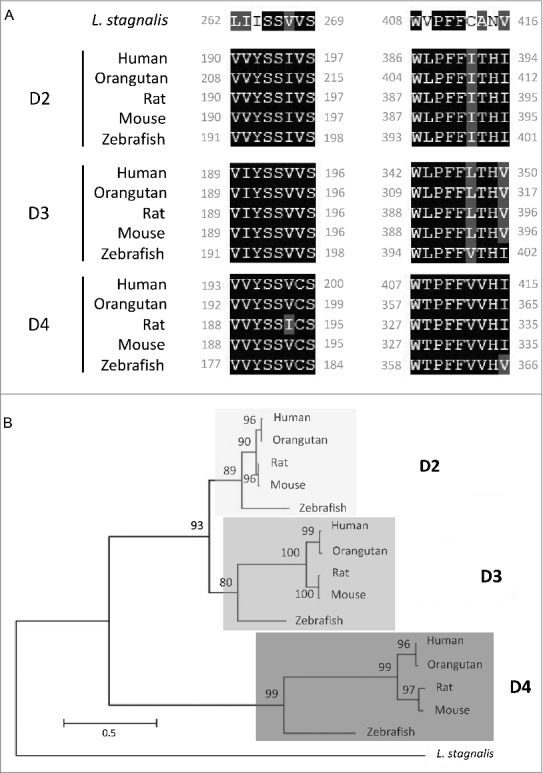
Protein sequence alignment and phylogenetic tree of the D2-like receptor in *L. stagnalis.* *A*, Amino acid sequence comparisons of the two agonist bindings sites between the single *L. stagnalis* D2-like receptor and the three isoforms of the D2-like subfamily of DA receptors in selected vertebrate species. Conserved and similar sequences in alignments are colored as black and grey boxes, respectively. *B*, Phylogenetic analysis was conducted by the Maximum Likelihood method using the JTT+G+I model. Bootstrap value from 500 replicates are shown next to each node. The scale bars indicate the estimated evolutionary distance in the units of the number of amino acid substitutions per site.

We first examined the *L. stagnalis* CNS transcriptome [82] to characterize the conservation of genes involved in DAergic regulation of Cav2 channels in central neurons. Whereas the D2-like subfamily of receptors in vertebrates is known to include three isoforms, D2, D3 and D4 receptors, we found that the *L. stagnalis* CNS expresses a single D2-like receptor (Figure 1) in which the two agonist bindings sites show partial sequence similarity with vertebrate D2-4 receptors. Consistent with previous reports [62,61], we observed that *L. staganalis* Gαi (Figure 2A) and Gαo (Figure 2B) subunits share high degrees of sequence identity with their vertebrate counterparts, with complete conservation of the four guanine nucleotide binding sites required for GTP binding. Similarly, the seven WD repeat domains in the single *L. stagnalis* Gβ protein (Figure 2C), which are implicated in protein-protein interaction [63], are nearly identical to those in vertebrate Gβ_1_ orthologs. Greater sequence divergence was observed in the single *L. stagnalis* Gγ isoform as compared to vertebrate Gγ_2_ orthologs (Figure 2D). Finally, sequence analyses of the *L. stagnalis* Ca_V_2 (*L*Ca_V_2) channel ortholog Ca_V_2, demonstrate that two of the three Gβ_1_γ_2_ binding sites, one in the N-terminus cytoplasmic tail and another in the I-II linker region, on vertebrate Ca_V_2.1 and Ca_V_2.2 channels that are essential for G protein-mediated voltage-dependent regulation of Cav2 channels [102] are conserved in considerable degrees in L Ca_V_2. Taken together, these findings demonstrate *L. stagnalis* to be a conserved model for examining the fundamental mechanisms of G protein-mediated DAergic modulation of Ca_V_2 channels in the CNS.

**Figure 2.**
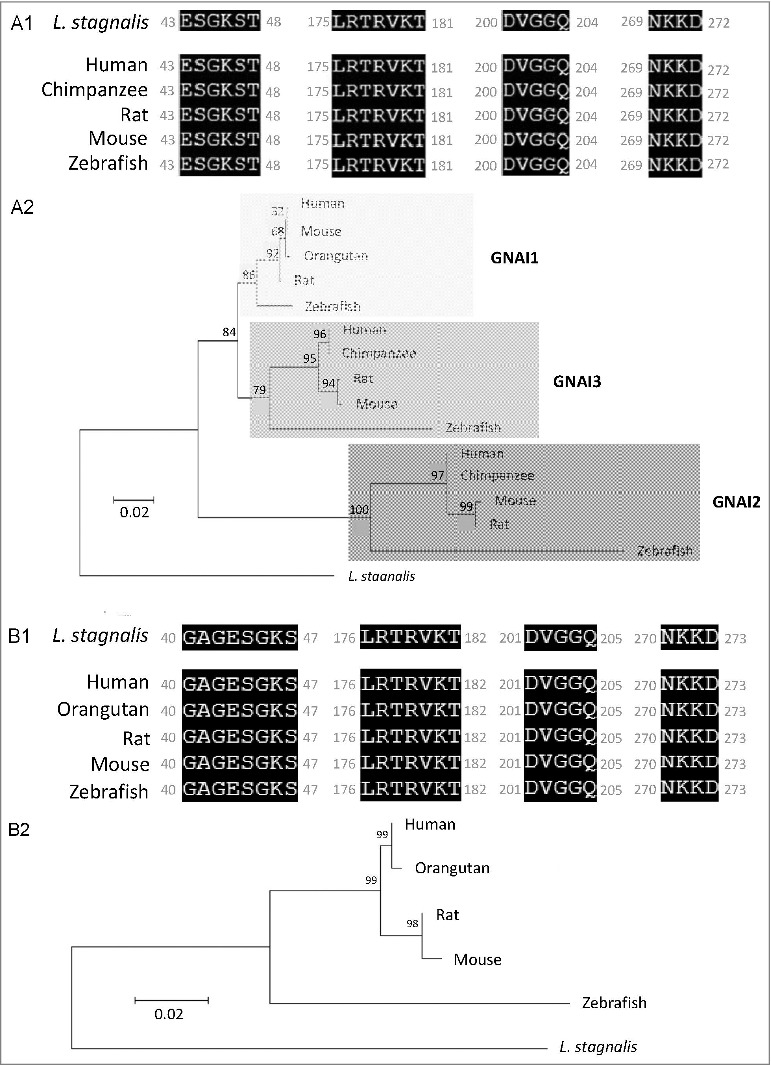
Protein sequence alignment and phylogenetic tree of G protein subunits in *L. stagnalis*. *A-B*,Amino acid sequence comparisons of the four nucleotide binding sites in the single *L. stagnalis* Gαi and Gαo orthologs with the vertebrate Gαi isoforms GNAI1-3 (A1) and Gαo (B1), respectively. As all four sites are identical across GNAI1-3 isoforms in human, chimpanzee, rat, mouse and zebrafish, only one instance is shown for simplicity. Phylogenetic analysis was conducted by the Maximum Likelihood method using the JTT+G model for Gαi (A2) and the LG+G model for Gαo (B2). *C*, Amino acid sequence comparisons of the seven WD repeat regions (WD1-7) of the single isoform *L. stagnalis* Gβ and vetebrate orthologs of Gβ_1_ (C1). Phylogenetic analysis was conducted by the Maximum Likelihood method using the WAG model (C2). *D*, Amino acid sequence comparisons of the single *L. stagnalis* Gγ isoform and vetebrate orthologs of Gγ_2_ (D1). Phylogenetic analysis was conducted by the Maximum Likelihood method using the LG model (D2). Conserved and similar sequences in alignments are colored as black and grey boxes, respectively. Bootstrap value from 500 replicates are shown next to each node. The scale bars indicate the estimated evolutionary distance in the units of the number of amino acid substitutions per site.

**Figure 2.**
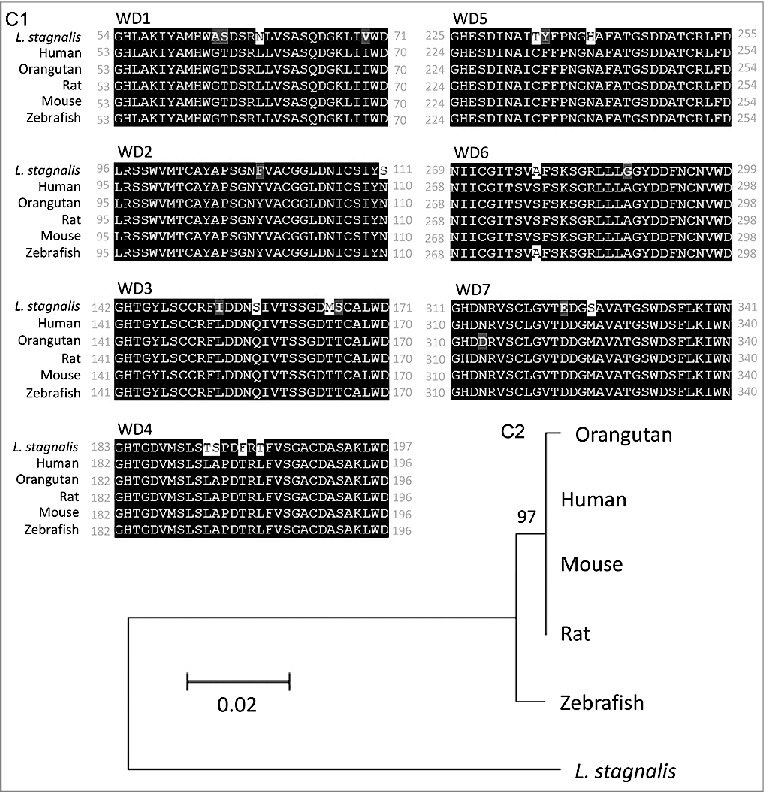
(Continued).

**Figure 2.**
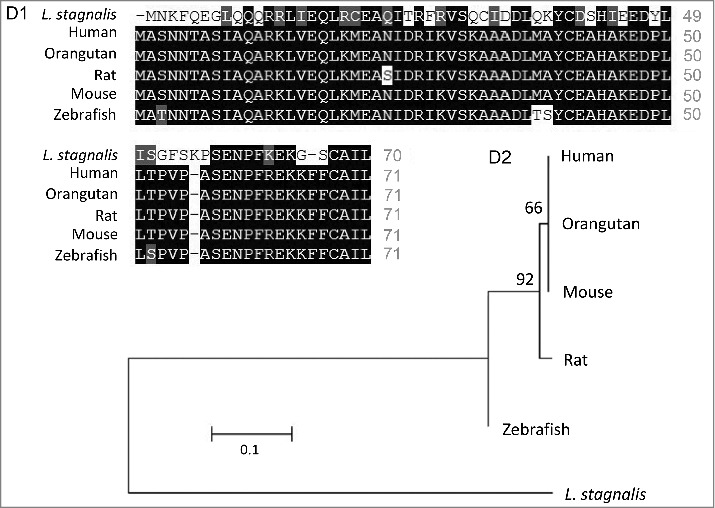
(Continued).


### DA inhibits synaptic output of the VD4

VD4 and LPeD1 were acutely isolated and cultured for 24 hours to allow for soma-soma synapse formation prior to testing, as previously described by [34]. Only pairs adopting the soma-soma synapse morphology were selected for recordings (Figure 4A). To test the effects of DA on synaptic transmission between the VD4 and LPeD1, postsynaptic responses to presynaptic APs were examined using dual-clamp recordings (Figure 4B). As it has previously been shown that 10 µM DA nearly abolishes all evoked action potential firing activity in VD4 interneurons [4], we employed 1 µM DA here to ensure sufficient VD4 synaptic output and detectable LPeD1 post-synaptic response. Indicative of the formation of an excitatory synapse between VD4 and LPeD1, recordings showed a one-to-one correlation between presynaptic APs and EPSCs (Figure 4C). As commonly observed, the first EPSC from the train of five APs had a larger amplitude than the remaining four APs. We noted that DA affected the properties of the AP train, however did not change the first AP of the train. We thus compared the first EPSC elicited by the first AP, before and after DA application. We found that DA resulted in a 37.8% reduction in peak amplitude of the first EPSC (no DA: −126.15 ± 7.97 pA; DA: −78.46 ± 10.38 pA, n = 4, *p* < 0.05) (Figure 4D). Inhibition of synaptic transmission by DA was reversed by washing with NSS bath solution (−101.33 ± 9.67 pA, n = 4).

We next sought to determine whether the DA-mediated reduction in EPSC amplitude was due to changes in vesicle release from the VD4, using the fluorescent styryl dye FM1-43 to visualize synaptic vesicle exocytosis (Figure 4E). FM1-43 labelled synaptic pairs show intensified labelling at the presynaptic side of the contact site (CS) before (left panel) and after (right panel) simulation (Figure 4E), indicating the location of the presynaptic recyclable vesicle pool. Exocytosis of FM1-43 labeled vesicles was elicited by a 5s burst of APs induced presynaptically. DA application resulted in a 70.2% (no DA: −4.7 ± 0.8 a.u.; DA: −1.4 ± 0.4 a.u.; n = 5; *p* < 0.05) reduction in FM1-43 destaining, which could be rescued by 36.2% after wash with NSS bath solution (-3.0 ± 0.6 a.u., n = 5; Figure 4F). Fewer vesicles situated at the pre- and postsynaptic non-contact sites (non-CSs) were released in response to evoked APs and their release was insensitive to DA (Figure 4F). Taken together, these findings indicate that DA inhibited synaptic output of the VD4 through suppression of vesicle release.

### DA inhibits *I*_Ca_ in the VD4

Activity-induced Ca^2+^ influx is a key determinant of presynaptic vesicle release. We first confirmed and expanded upon earlier findings by [4]. that 10 µM DA indeed inhibited the amplitude of *I*_Ca_ in the VD4 (Figure 5A and B), without affecting its activation properties (Figure 5C). However, as a non-physiological square wave (SW) protocol was used to evoke *I*_Ca_, the physiological relevance of the observed inhibition by DA was unclear. Therefore, as shown in Figure 6A, we employed pre-recorded VD4 AP waveforms (APW) as the voltage clamp command to examine the effect of the same concentration of DA on intracellular Ca^2+^ dynamics during neuronal activity. We then compared the *I_Ca_* peak amplitude induced by either APW (Figure 6B) or SW (Figure 6C) in response to 10 μM DA. We found that DA reduced the average peak amplitude of APW-evoked *I*_Ca_ by 36.9 ± 9.01% (n = 4) (Figure 6B), which was comparable with the extent of DA-mediated reduction observed in *I*_Ca_ elicited by a SW voltage step to peak APW amplitude (+30 mV) (36.10 ± 5.03%, n = 6; p>0.05) (Figure 6C). The 10 µM DA-induced reduction of I_Ca_ evoked by SW or APW DA was consistent, as summarized in Figure 6D. Clearly, DA inhibited Ca^2+^ influx evoked by neuronal activity in the VD4.

### Prepulse depolarization facilitates *I*_Ca_ in the presence of DA

Next, we sought to investigate the mechanisms underlying DAergic inhibition of *I*_Ca_ in the VD4. GPCR-mediated inhibition of presynaptic voltage-gated Ca^2+^ channels, via the liberated Gβγ subunit, can be detected using a prepulse depolarization that causes unbinding of the Gβγ subunit from the channel and disinhibition of *I*_Ca_ [47,54] (Figure 7A). We found that DAergic inhibition of *I*_Ca_ (36.10 ± 5.03%, n=5) in the VD4 was significantly reduced by prepulse depolarization (26.95 ± 3.41%, n=5, *p* < 0.05) (Figure 7B and C). The prepulse had no effect on *I*_Ca_ in the absence of DA (Figure 7B and C). These findings suggest that DAergic inhibition of *I*_Ca_ in the VD4 involved Gβγ-dependent signalling.

### DA inhibits ionomycin-induced synaptic vesicle release from the VD4

Several studies have shown that neuromodulators can also inhibit presynaptic vesicle release in neurons independently of their effects on Ca^2+^ dynamics, such as through direct G protein-dependent interaction with the exocytic machinery [70,11,12,40,41,78,93,100]. To investigate the possibility that DA also modulates VD4 synaptic release downstream of Ca^2+^ influx, we employed the ionophore ionomycin to mediate Ca^2+^ entry into the cell independently of voltage-gated Ca^2+^ channels [18]. Upon addition of ionomycin, the linear relationship between extracellular Ca^2+^ concentration and FURA-2 imaging of intracellular Ca^2+^ concentration indicated that the source of the Ca^2+^ influx is from the extracellular bath (Figure 8A). First, we confirmed that ionomycin-induced Ca^2+^ influx was sufficient to trigger vesicle release by monitoring intracellular Ca^2+^ concentration and vesicle release using FURA-2 fluorescence and FM1-43 destaining, respectively (Figure 8B). We observed that ionomycin caused a rapid influx of Ca^2+^ that then reached an equilibrium level and remained constant. In parallel, FM1-43 fluorescence traces showed two components: a rapid exponential decrease in fluorescence directly after ionomycin application, followed by a slow linear decay in fluorescence. Finally, we examined the effects of DA on ionomycin-induced vesicle release. Interestingly, exogenous DA was still able to attenuate ionomycin-induced vesicle release in the VD4 by 66.9% (no DA: 221.2 ± 35.6 a.u., n = 6; DA: 73.3 ± 11.5 a.u., n = 4; *p* < 0.05) (Figure 8C and D). Taken together, these results indicate that DA also inhibited VD4 synaptic output via voltage-gated Ca^2+^ channel-independent mechanisms.

### Discussion

In this study, we examined the effects of DA on *I*_Ca_ and vesicle release in a well-characterized molluscan interneuron synapse model. We demonstrated for the first time that DAergic inhibition of synaptic output is mediated through both voltage-dependent and -independent inhibition of voltage-gated Ca^2+^ channels, and voltage-gated Ca^2+^ channel-independent suppression of vesicle release.

### The DAergic system in *L. stagnalis*

In mammalian neurons, DA broadly regulates neurotransmission through activation of a family of GPCRs, which are divided into two major classes based on their structural, pharmacological, and signalling properties: D1 and D5 receptors belong to the subfamily of D1-like receptors, whereas D2, D3, and D4 receptors are grouped into the D2-like receptor class. Both D1/3- and D2/4-like receptors have been identified in *L. stagnalis* based on their pharmacological properties, albeit with differing sensitivities to various agonists and antagonists as compared to their vertebrate counterparts [86,3,48,69,94]. The D1- and D2-like receptors in *L. stagnalis* exhibit differential expression patterns, with the former present on growth cones and the latter on the soma [26]. The effects of DA on the VD4 are shown to be mediated by D2-like receptors, as it has been shown that the effets of both synaptically released DA from the RPeD1 and exogenously applied dopamine on the VD4 can be blocked *only* by sulpiride, a D2 receptor antagonist [68]. Indeed, DAergic inhibition of *I*_Ca_ in the VD4 [4] is consistent with observations of D2 receptor effects in mammalian CNS [74,16,50,73]. While the downstream targets and signaling mechanisms of D2-like receptors in *L. stagnalis* remain unclear, previous studies [62,61]; Knol *et al.*, 1995) and our sequence analyses of G protein subunits (Figure 2) in the *L. stagnalis* CNS have demonstrated that they are highly homologous to their vertebrate counterparts. Taken together, *L. stagnalis* provides a conserved model for studying DAergic regulation of interneuron function.

There are several potential limitations to the cultured VD4 interneuron electrophysiological experimental model employed in studies thus far, including our current report, in recapitulating the physiological conditions of DAergic modulation of interneurons in *L. stagnalis*. As the VD4 likely receives pulsatile DAergic input from the presynaptic rhythmically active pacemaker neuron RPeD1 [85,88], the bath-applied tonic DA treatment may not fully mimick the effects of phasically released DA on VD4 membrane and synaptic properties *in vivo.* While the sensitivity of cultured VD4 interneurons to bath-applied DA has been characterized [4], it is yet unclear what range of DA concentrations act on the VD4 interneuron *in vivo*.

### Voltage-dependent and independent inhibition of *I*_Ca_ by DA

Activity-induced Ca^2+^ influx is a key determinant of presynaptic vesicle release [104]. A reduction in the amount of Ca^2+^ influx through voltage-gated Ca^2+^ channels at the active zone would efficiently reduce the probability of synaptic vesicle fusion and therefore neurotransmission [58,46]. Previous studies have reported that DAergic inhibition of synaptic output in interneurons is associated with reductions in *I*_Ca_ in mammalian interneurons [99,16]. In this study, we confirmed and expanded upon the findings of [4]. by demonstrating that DA inhibits voltage-dependent Ca^2+^ influx elicited by physiologically relevant stimuli, i.e. action potential waveforms, in the VD4. These findings suggest that DA likely inhibits activity-induced Ca^2+^ influx in the presynaptic terminal that is required for neurotransmitter release.

**Figure 3.**
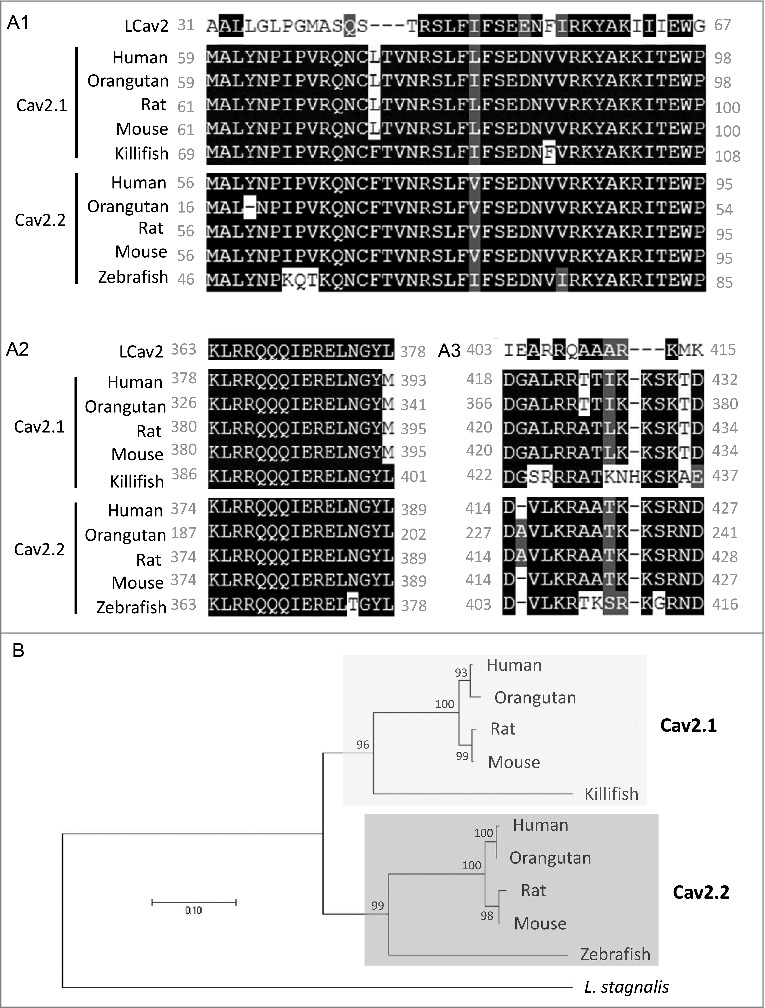
Protein sequence alignment and phylogenetic tree of the Cav2 channel in *L. stagnalis*. *A*, Amino acid sequence comparison of the Gβγ binding sites on the single *L. stagnalis* Cav2 channel isoform, LCav2, N-terminus cytoplasmic tail (*A1*) and I-II linker region (*A2-3*) with vertebrate orthologs of Cav2.1 and Cav2.2. Conserved and similar sequences in alignments are colored as black and grey boxes, respectively. Gaps are denoted by dashes. *B*, Phylogenetic analysis was conducted by the Maximum Likelihood method using the JTT+G model. Bootstrap value from 500 replicates are shown next to each node. The scale bars indicate the estimated evolutionary distance in the units of the number of amino acid substitutions per site.

Prepulse facilitation of VD4 *I*_Ca_ in the presence of DA indicates that this inhibition is mediated in part through voltage-dependent G-protein–mediated inhibition, which is shown to be mediated through direct binding of membrane-delimited Gβγ to cytoplasmic regions of the α1 subunit of Ca_V_2 family of voltage-gated Ca^2+^ channels, resulting in delay of the activation kinetics and a depolarizing shift in the activation voltage of the channel [27,5,31,36,47,54,103]. In this study, we observed only a reduction in *I*_Ca_ amplitude without changes in its activation kinetics. As the effects of Gβγ-mediated inhibition are shown to be dependent on the Ca_V_ β subunit subtype [17,32], this may in part be due to differences in the *L. stagnalis* and mammalian Ca_v_ β subunit properties. A previous study reported that the *L. stagnalis* Ca_V_2 ortholog, *L*Ca_V_2, does not exhibit prepulse facilitation when co-expressed in HEK-293T cells with rat orthologs of the Ca_V_2 channel accessory subunits α2δ1 and β1b and G protein subunits β1 and γ2 [51]. As protein-protein interaction between LCa_V_2 and rat Gβ1γ2 was not directly confirmed in the study, and given the sequence divergence we observed between *L. stagnalis* and vertebrate Gβ (Figure 2C), Gγ (Figure 2D), and Ca_V_2 channel Gβγ binding sites (Figure 3), the discrepancies between our findings may be attributable in part to non-canonical associations between LCa_V_2 and mammalian Gβγ protein. In addition, endogenous factors present in *L. stagnalis* neurons but absent in HEK-293T cells may also be required for the G protein-mediated voltage-dependent regulation of LCa_V_2 channels observed in this study.

**Figure 4.**
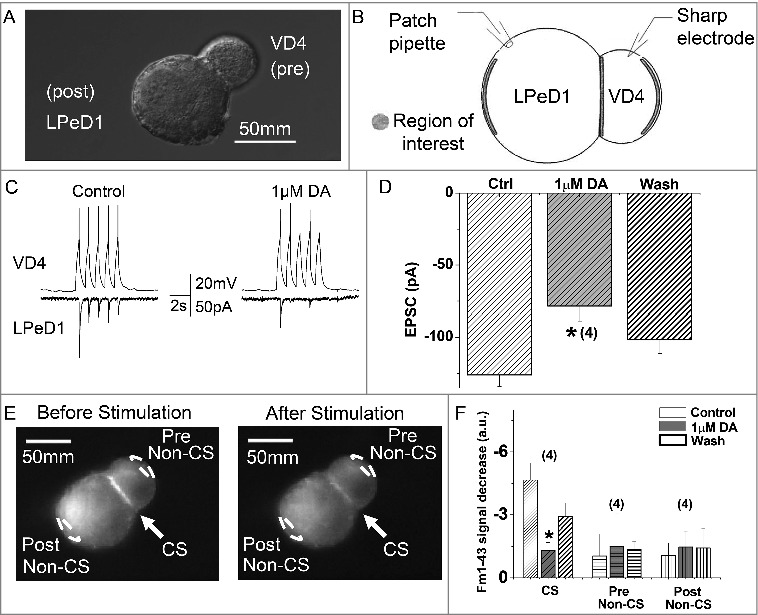
DA inhibits vesicle release from VD4 interneurons. *A*, DIC image of a soma-soma synaptic pair. VD4 and LPeD1 were isolated and placed in culture for 24 hours to allow for soma-soma synapse formation. *B*, Schematic of the dual-clamp configuration to simultaneously monitor pre- and post-synaptic activity in synaptically paired VD4 and LPeD1. *C*, Representative traces of simultaneous pre-synaptic action potentials (APs) recoreded from VD4 cell and excitatory post-synaptic currents (EPSCs) recorded from LPeD1 cells, before (left) and after (right) DA application. *D*, Summary of the amplitude of the first EPSCs recorded from LPeD1 cells, before (Ctrl), during (1 µM DA) and after (Wash) dopamine application. *E*, Representatives of FM1-43 imaging showing distribution of FM1-43 staining with high intensity at the cell-contact region (arrow, CS), as well as the sites of non-contact site (white dashed line ovals) at the pre- (Pre Non-CS, VD4 cell) and post-synaptic cells (Post non-CS, LPeD1 cell) respectively. FM1-43 labelling before (left panel) and after (right panel) action potential stimuli in VD4 cells. The reduction in fluorescence intensity at CS indicates the presynaptic vesicle release. *F*, Summary of the changes in FM1-43 signal at the different regions indicated in *E*, showing DA inhibits vesicle release from the VD4 at the CS, but not at non-CSs. The patterns of the bars showing the sites of the images were taken, and the sequence of the bars with different background color shows the recording conditions, in order of control (first bar, white), 1 uM DA (second bar, grey) and wash (third bar, white), at indicated sites where fluorescent intensities were taken. *p < 0.05.

**Figure 5.**
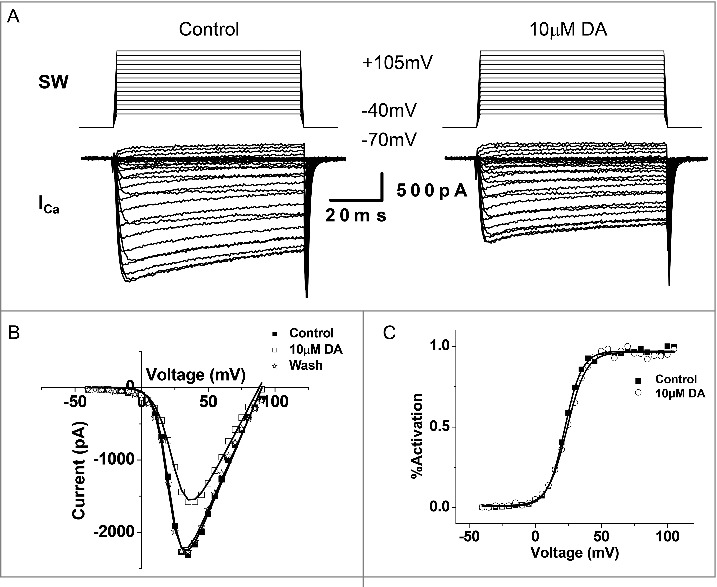
DA inhibits *I*_Ca_ in VD4 interneurons. *A*, Whole cell voltage clamp recordings were performed in VD4 interneurons to characterize the effects of DA on *I*_Ca._ Representative traces of *I*_Ca_ elicited by SW voltage steps in the VD4 before (left) and after (right) DA application. *B*, Representative IV relationship of peak *I*_Ca_ before and after DA application, and after washout. *C*, Representative activation curves of *I*_Ca_ before and after DA application.

**Figure 6.**
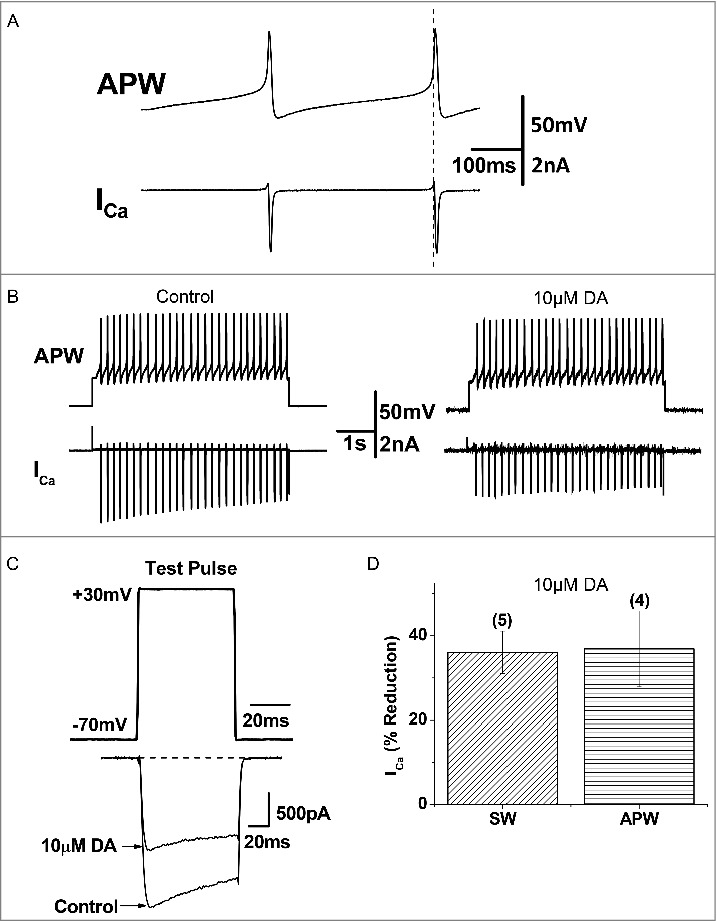
DA inhibits action potential waveform (APW)-evoked *I*_Ca_ in VD4 interneurons_._ *A*, To mimic physiologically relevant neuronal activity, APs recorded from a VD4 interneuron were used to elicit *I*_Ca_ in whole cell voltage-clamped cultured VD4 interneurons. Upper panel: APWs recorded from VD4 cells were used as the stimulus protocol. Lower panel: the inward I_Ca_ recorded in response to each APW stimulus. *B*, Representative traces of APW-evoked *I*_Ca_ in the absence or presence of DA. *C*, Representative traces of *I*_Ca_ evoked by a square-waveform (SW) voltage step from the resting membrane potential of −70 mV to +30 mV, the APW peak amplitude (+30mV), in the absence or presence of DA. *D*, Comparable reductions are observed in SW- and APW-evoked *I*_Ca_ in the presence of DA. * *p* < 0.05.

**Figure 7.**
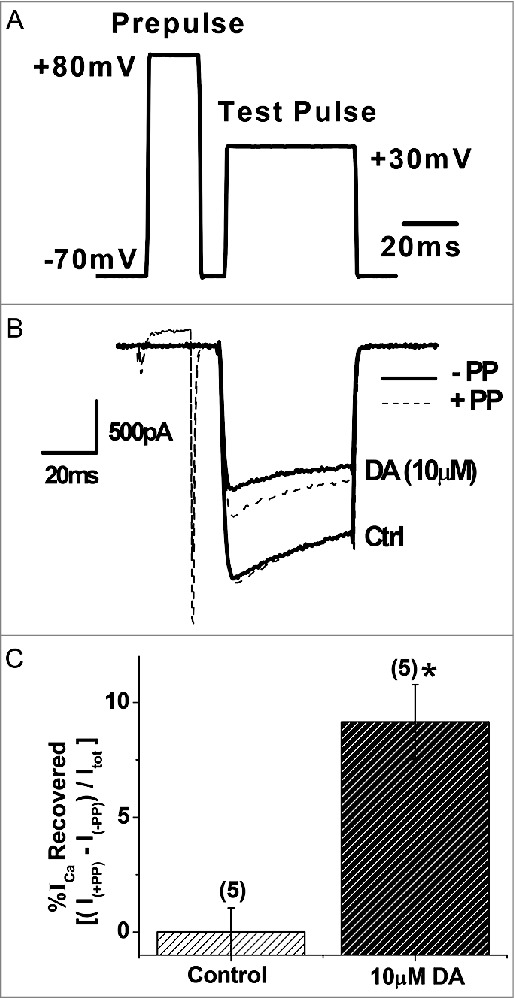
Prepulse facilitation of *I*_Ca_ in VD4 interneurons in the presence of DA. *A*, The prepulse protocol employed in whole cell voltage clamp recordings of VD4 interneurons. *B*, Representative traces of *I*_Ca_ evoked by the test pulse without (solid line) and with (dashed line) a prepulse, in the absence or presence of DA. *C*, Significant recovery of *I*_Ca_ by prepulse depolarization is observed only in the presence of DA. * p < 0.05.

**Figure 8.**
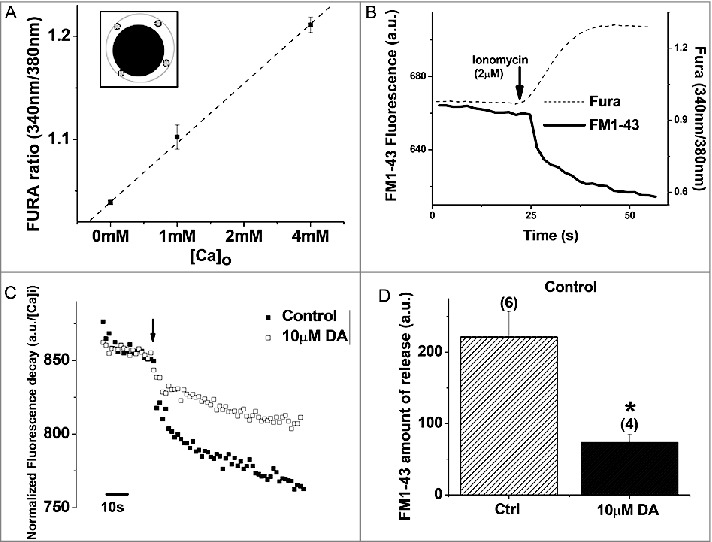
DA inhibits vesicle release from VD4 interneurons downstream of Ca^2+^ influx. *A*, Ionomycin-evoked Ca^2+^ influx in the VD4 interneuron, as measured by averaged FURA-2 signal from four proximal-membrane regions (inset), is proportional to extracellular Ca^2+^ concentration ([Ca]_o_). *B*, Ionomycin-induced Ca^2+^ influx, as measured by FURA-2 signal (grey), is sufficient to induce vesicle release, as indicated by FM1-43 destaining (black). *C*, Representative FM1-43 destaining curve upon ionomycin application (arrow) in the absence (filled) or presence (open) of DA. *D*, Ionomycin-induced vesicle release from VD4 interneurons is significantly reduced in the presence of DA. * *p* < 0.05.

The depolarizing prepulse relieved only ∼30% of DAergic inhibition of *I*_Ca_ in the VD4 in our study, suggesting that DA also inhibited *I*_Ca_ through a voltage-independent mechanism. G protein-mediated voltage-independent inhibition of LCa_V_2 in the VD4 has been reported to be mediated through the Gα subunit and increases in cAMP levels, with a slow time course on the scale of minutes [51]. Slow but persistent voltage-independent G protein-mediated inhibition of voltage-gated Ca^2+^ channels has also been well-documented in mammalian neurons [9,6,7,22,56,66,83,87,102] and shown to be mediated by a variety of mechanisms, including regulation of Ca_v_ surface expression levels through direct interaction with GPCRs [59,60] and phosphatidylinositol 4,5-bisphosphate (PIP_2_) depletion [49,38] and kinase activation [80,25].

Parallel activation of voltage-dependent and independent inhibition of voltage-gated Ca^2+^ channels has been hypothesized to allow for fine-tuning of intracellular Ca^2+^ levels and activity-dependent plasticity [66,14,51,76,95]. The relative ratio between voltage-dependent and -independent modes of inhibition in chick sensory neurons is shown to be critically dependent on intracellular Gβγ concentration [64]. Activation of D2 receptors induces both voltage-dependent and -independent inhibition of the N-type voltage-gated Ca^2+^ channels in HEK-293T cells [60] and differentiated NG108-15 cells [15]. Consistent with the previous reports, our findings demonstrated DA effects are mediated by both voltage-dependent and -independent inhibition. It is likely that the synaptically released DA level onto interneurons varies, and thus can differentially modify interneuron intracellular Ca^2+^ dynamics and synaptic output, thereby inducing cellular and circuit-wide plasticity.

### DAergic inhibition of vesicle release downstream of Ca^2+^ influx

In neurons and secretory cells, GPCRs can inhibit vesicle release in parallel with changes in *I*_Ca_ [70,18,24,77,84]. This inhibition can occur through direct binding of the Gβγ subunit to the C-terminus of the SNARE protein SNAP-25, thereby displacing synaptotagmin and preventing vesicle exocytosis [40,93,105]. Gβγ is also known to interact with several other proteins involved in synaptic vesicle release, including syntaxin, VAMP, CSP and synaptotagmin [55]. Binding of Gβγ to these proteins may alter their function and/or interaction with other proteins in the exocytic machinery, thereby inhibiting vesicle release downstream of Ca^2+^ influx [90]. While no direct interaction between G protein and presynaptic proteins have been reported in *L. stagnalis*, they are shown to be homologous in structure and function to their vertebrate counterparts (Figure 2) [62,61]; Knol *et al.*, 1995; [35,43]. Here we employed the ionophore ionomycin to induce Ca^2+^-dependent synaptic release independently of voltage-gated Ca^2+^ channel activation, as it has been shown in hippocampal neurons that ionomycin-induced synaptic release is unaffected by the voltage-gated Ca^2+^ channel blocker Cd^2+^ [18]. We demonstrated that DA inhibits vesicle release from VD4 interneurons downstream of activity-induced Ca^2+^ influx. Our finding suggests that *L. stagnalis* may serve as an easily tractable model in which to elucidate the molecular mechanisms of G protein-mediated modulation of synaptic release.

### Potential functional significance of redundant mechanisms of DAergic inhibition of synaptic transmission in interneurons

In this study, we provide the first demonstration that inhibition of *I*_Ca_ and direct inhibition of synaptic vesicle release all contribute to DAergic presynaptic inhibition in interneurons. It may be hypothesized that whereas the Gβγ subunit-mediated voltage-dependent inhibition allows for rapid activity-dependent regulation of synaptic transmission, the voltage-independent inhibition of *I*_Ca_ and direct inhibition of vesicle release machinery may allow for more persistent and nuanced inhibition of interneuron synaptic output [66,14,51,76,95]. Indeed, strong but modifiable inhibition of the VD4 activity may be integral to respiratory control in *L. stagnalis*. In the context of the respiratory CPG network, the VD4 controls the muscles that close the pneumostome [89]. Increased firing of the RPeD1 neuron, which results in increased DA release onto VD4, coincides with opening of the pneumostome and initiation of the inspiration phase [88]. Therefore, complete inhibition of VD4 output during this phase is likely crucial to prevent interruption of inspiration and thus generating normal rhythmic respiration behaviour. In addition, the capacity for activity-dependent plasticity in the VD4 response to DA, endowed by voltage-dependent inhibition of voltage-gated Ca^2+^ channels, may be important for the well-documented hypoxia and learning-induced modulation of CPG activity and aerial respiration behaviour in *L. stagnalis* [67,8].

### Conclusion

In this study, we provide the first demonstration that DA suppresses synaptic output of a molluscan respiratory interneuron through both voltage-dependent and -independent inhibition of *I*_Ca_, and voltage-gated Ca^2+^ channel-independent inhibition of synaptic vesicle release. In future studies, the relative sensitivity and function of each pathway and any potential interactions between them will be further investigated to expand our understanding of the fundamental mechanisms that shape interneuron function and neuronal network dynamics.
